# Inhibition of HIF-1alpha activity by homeodomain-interacting protein kinase-2 correlates with sensitization of chemoresistant cells to undergo apoptosis

**DOI:** 10.1186/1476-4598-8-1

**Published:** 2009-01-07

**Authors:** Lavinia Nardinocchi, Rosa Puca, Ada Sacchi, Gabriella D'Orazi

**Affiliations:** 1 Department of Experimental Oncology, Molecular Oncogenesis Laboratory, Regina Elena Cancer Institute, 00158 Rome, Italy; 2 Department of Oncology and Neurosciences, University "G. D'Annunzio", 66013 Chieti, Italy

## Abstract

**Background:**

Homeodomain-interacting protein kinase-2 (HIPK2), a transcriptional co-repressor with apoptotic function, can affect hypoxia-inducible factor 1 (HIF-1) transcriptional activity, through downmodulation of its HIF-1α subunit, in normoxic condition. Under hypoxia, a condition often found in solid tumors, HIF-1α is activated to induce target genes involved in chemoresistance, inhibition of apoptosis and tumor progression. Here, we investigated whether the HIPK2 overexpression could downregulate HIF-1α expression and activity in tumor cells treated with hypoxia-mimicking condition, and evaluated whether HIPK2-dependent downregulation of HIF-1α could sensitize chemoresistant tumor cells to adriamycin (ADR)-induced apoptosis.

**Methods:**

Tumor cell lines carrying wild-type p53, siRNA p53, or mutant p53 were overexpressed with HIPK2 (full length or catalytic inactive mutant) and treated with cobalt chloride (CoCl_2_) to mimic hypoxia, in the presence or absence of ADR treatment. HIF-1α expression was measured by semiquantitative reverse-transcriptase (RT)-PCR and Western immunoblotting and HIF-1 activity was evaluated by luciferase assay using reporter plasmid containing hypoxia response elements (HREs) upstream of luciferase gene. HIF-1 target genes, including multidrug resistance 1 (MDR1) and the antiapoptotic Bcl2 were determined by RT-PCR. Cell survival and apoptosis were measured by colony assay and cleavage of the caspase-3 substrate PARP, respectively.

**Results:**

Overexpression of HIPK2 resulted in downmodulation of cobalt-stabilized HIF-1α protein and HIF-1α mRNA levels, with subsequent inhibition of HIF-1 transcriptional activity. MDR1 and Bcl-2 gene expression was downmodulated by HIPK2 overexpression in cobalt-treated cells. Inhibition of HIF-1 transcriptional activity was dependent on HIPK2 catalytic activity. HIPK2 overexpression did not induce per se apoptosis of cobalt-treated cells, on the contrary it sensitized cobalt-treated cells to ADR-induced apoptosis, regardless of their p53 status.

**Conclusion:**

The ability of HIPK2 to restore the apoptosis-inducing potential of chemotherapeutic drug in hypoxia-mimicking condition and therefore to sensitize chemoresistant tumor cells suggests that HIPK2 may induce fundamental alterations in cell signaling pathways, involving or not p53 function. Thus potential use of HIPK2 is promising for cancer treatment by potentiating cytotoxic therapies, regardless of p53 cell status.

## Background

Homeodomain-interacting protein kinase-2 (HIPK2) is a serine/threonine kinase [1] that as been shown to be involved in restraining tumor progression. HIPK2 carries out its potential oncosuppressor function through both its catalytic and transcriptional co-repressor activities. We and others have found that HIPK2 phosphorylates p53 at serine 46 (Ser46) after severe DNA damage and induces apoptosis [2,3]; we also found that silencing of HIPK2 inhibits p53 transcriptional activity [4], inhibits drug-induced apoptosis inducing chemoresistance [5], and increases genomic instability [6], moreover, we showed that HIPK2 ovrexpression circumvents the blockade of apoptosis in cisplatin-resistant cells, restoring the p53-dependent apoptosis [7]. The role of HIPK2 in regulating tumor growth has been also evidenced in p53-independent manner. We have shown that HIPK2 downregulates the cPLA2-induced prostaglandin E_2 _(PGE_2_) production, and that HIPK2 silencing increases *in vivo *tumor growth and tumor vascularity [8,9]. Thus, we have found that HIPK2 can suppress the β-catenin-mediated transcriptional activation of vascular endothelial growth factor (VEGF) [10]. Furthermore, we have recently found that HIPK2 binds to the hypoxia-inducible factor 1α (HIF-1α) promoter, in a co-repressor complex with histone deacetylase 1 (HDAC1), to repress HIF-1α at transcriptional level with subsequent suppression of HIF-1 transcriptional activity, in normoxic condition [9].

HIF-1 is a heterodimeric transcription factor that transactivates more than 60 target genes involved in multiple aspects of tumorigenesis including tumor growth, angiogenesis, metastasis, and chemotherapy response [11]. HIF-1 consists of two subunits, HIF-1α and HIF-1β [12]: HIF-1β is constitutively expressed in cells, while HIF-1α stability is stimulated by hypoxia, growth factors, and several oncogenes [13] thus, HIF-1α is overexpressed in many types of tumors and contributes to chemoresistance [14]. Activation of HIF-1 depends primarily upon redox-sensitive stabilization of its α subunit [15] however, HIF-1 responsive genes can be induced by cobalt ions with kinetics similar to that of hypoxia induction [16].

The mechanisms of chemoresistance appear to be multifactorial and include, among others, inhibition of apoptosis and dysfunction of p53 oncosuppressor [17]. Growing evidence suggests that hypoxia in tumors selects for cells with decreased potential for apoptosis through for instance the overexpression of the antiapoptotic gene Bcl-2 and decreased killing effect through upregulation of drug transporter proteins that may contribute to resistance to standard radio- and chemo-therapy [11,18]. Thus, multidrug resistance (MDR) is a serious obstacle for cancer chemotherapy [19]. The MDR phenotype is associated with induction of the *MDR1 *gene [20] which encodes for P-glicoprotein a transporter protein able to extrude a range of anti-cancer drugs out of the cell, lowering their concentration below the level required for cytotoxic effects [14]. *MDR1 *has been shown to be HIF-1 target gene [21] and is involved in the acquisition of multidrug resistance in various types of cancers cells, for instance, tumors resistance to doxorubicin is associated with increased levels of *MDR1 *gene [19].

A recent survey of malignant and normal tissues found that the expression of HIF-1α is commonly increased in a variety of human tumors including colorectal carcinoma and glioblastoma [22]. The purpose of our investigation was to evaluate, in colon cancer and glioblastoma cell lines, whether HIPK2 might inhibit HIF-1α expression and activity in hypoxia-mimicking condition and sensitize chemoresistant tumor cells to adriamycin (ADR)-induced apoptosis. The results show that overexpression of HIPK2 inhibited HIF-1α mRNA levels and cobalt-stabilized HIF-1α protein, with subsequent suppression of HIF-1 transcriptional activity. The HIPK2-mediated inhibition of HIF-1α correlated with suppression of MDR1 and Bcl-2 gene transcription and with sensitization of cobalt treated-tumor cells, regardless of their p53 status, to ADR-induced apoptosis. Thus, HIPK2 could be useful in cancer therapy to enhance the effectiveness of chemotherapeutic drugs.

## Results

### HIPK2 downregulates HIF-1α expression and protein levels in hypoxia-mimicking condition

**Figure 1:**
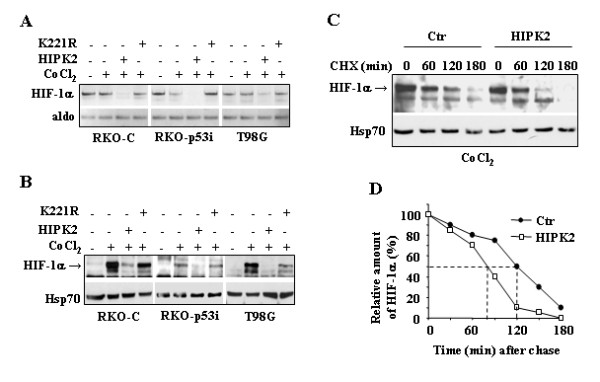
**HIPK2 downregulated HIF-1a expression and protein levels in hypoxia-mimicking condition**. (A), RKO-C, RKO-p53i, and T98G cells were transfected with HIPK2-GFP or K221R-GFP expression vectors. Twelve hours after transfection cells were treated with CoCl_2 _(200 μM) for 24 h. Thirty-six hours after transfection cells were harvested and HIF-1α expression was determined by RT-PCR analysis (Total time treatments: 36 h transfection, 24 h CoCl_2_). Aldolase (aldo) was used as loading control. One representative experiment from three independent experiments is shown. (B), Nuclear extracts from cells treated as in (A) were analyzed by Western immunoblotting with anti-HIF-1α antibody. Anti-Hsp70 was used as protein loading control. (C), RKO-C cells were transfected with HIPK2-GFP or GFP-empty vectors. Twelve hours after transfection cells were treated with CoCl_2 _(200 μM) for 20 h before adding cycloheximide (CHX) (40 μg/ml, t = 0). Nuclear extracts were analyzed by Western immunoblotting 60, 120, and 180 minutes following CHX treatment, with anti-HIF-1α antibody. Anti-Hsp70 was used as protein loading control. (D), The relative amount of HIF-1α protein levels from panel (C) was quantitated by densitometric analysis and plotted. The half-life of HIF-1α is shown by dotted lines.

To address whether HIPK2 might regulate the HIF-1α expression induced by hypoxia, tumor cells were treated with cobalt chloride (CoCl_2_) that stabilizes HIF-1α and induces HIF-1 responsive genes with kinetics similar to that of hypoxia [16]. RKO colon cancer cells, carrying wild-type p53, stably transfected with siRNA control vector (RKO-C) or with siRNA p53 vector (RKO-p53i), and T98G glioblastoma cells, carrying mutant p53, were transfected with HIPK2 or its catalytic inactive mutant K221R. After transfection, cells were treated with CoCl_2 _(200 μM) for 24 h. As shown in Fig. 1A, HIPK2 overexpression downmodulated HIF-1α mRNA levels while the K221R mutant failed to do so. HIF-1α mRNA expression levels were not induced by cobalt treatment, as expected. Furthermore, the cobalt-stabilized HIF-1α protein levels were strongly suppressed by HIPK2 overexpression, compared to the K221R kinase inactive mutant in all three cell lines analyzed (Fig. 1B). Then, the HIF-1α protein half-life was evaluated after HIPK2 overexpression. RKO-C cells were transfected with either HIPK2 expression vector or the empty vector and 24 h later exposed to cobalt treatment to induce HIF-1α accumulation. Thereafter (t = 0), the synthesis of HIF-1α protein was inhibited by the addition of cycloheximide (CHX) and the degradation of the cobalt-stabilized HIF-1α protein was monitored for a further period of 180 min. As shown in Fig. 1C, HIPK2 was able to accelerate the degradation of cobalt-stabilized HIF-1α, compared to the empty vector-transfected cells. Densitometric analysis of protein expression show that the HIF-1α half-life after transfection with the empty vector was of about 120 min and drop down to about 80 min after HIPK2 overexpression (Fig. 1D). These data suggest that HIPK2 downmodulated HIF-1α mRNA and protein levels under hypoxia-mimicking condition and that the HIPK2 catalytic activity was involved in this regulation.

### HIPK2 inhibited the HIF-1α transcriptional activity of target genes

**Figure 2:**
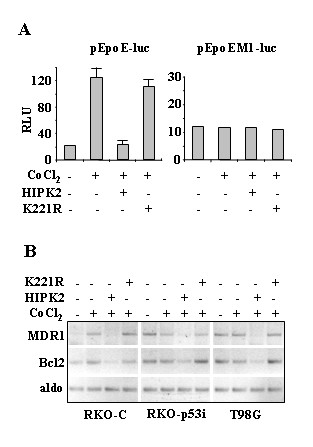
**HIPK2 inhibited HIF1 transcriptional activity of target genes**. (A), H1299 cells were transiently co-transfected with HIPK2-GFP or K221R-GFP expression vectors along with the luciferase reporter gene driven by human erythropoietin enhancer region containing a functional HIF-1 binding site (pEpoE-luc) or a mutant HIF-1 binding site (pEpoEM1-luc). Twelve hours after transfection cells were treated with CoCl_2 _(200 μM) for 24 h. Thirty-six hours after transfection luciferase activity was assayed. Relative Luciferase Units (RLU) normalized to β-gal is shown. The shown data represent the mean ± SD from three independent experiments performed in duplicate. (B), RKO-C, RKO-p53i, and T98G cells were transfected with HIPK2-GFP or K221R-GFP expression vectors. Twelve hours after transfection cells were treated with CoCl_2 _(200 μM) for 24 h. Thirty-six hours after transfection cells were harvested and MDR1 and Bcl2 expression was determined by RT-PCR analysis. Aldolase (aldo) was used as loading control. One representative experiment from three independent experiments is shown.

Next, the function of HIF-1 pathway was investigated by using a transient reporter assay in which endogenous HIF-1 binds hypoxia response elements (HREs) of erythropoietin gene promoter cloned upstream of a luciferase transcriptional reporter (pEpoE-luc); a vector containing the mutated HIF-1 binding site (pEpoEM1-luc) was also evaluated [15]. To this end, H1299 cells were co-transfected with pEpoE-luc or pEpoEM1-luc reporters and HIPK2 or K221R expression vectors. After transfection cells were treated with CoCl_2 _for 24 h. As shown in Fig. 2A, overexpression of HIPK2 markedly suppressed the cobalt-induction of luciferase gene expression. Moreover, this effect correlated with the HIPK2 catalytic activity. HIPK2 did not affect the activity of the pEpoEM1-luc reporter that was neither induced by cobalt-treatment (Fig. 2A). Thus, the effect of HIPK2 on luciferase gene expression was HIF-1-dependent.

Next we evaluated whether HIPK2 could suppress HIF-1 target gene transcription. To this aim, RKO-C, RKO-p53i, and T98G cells were transfected with HIPK2 or K221R and soon after transfection treated with CoCl_2 _for 24 h. Semiquantitative RT-PCR analysis of mRNA levels showed that the MDR1 and Bcl2 gene induction in cobalt treated-RKO-C cells was strongly suppressed by HIPK2 overexpression but not by K221R mutant (Fig. 2B). On the other hand, the already elevated MDR1 and Bcl2 mRNA levels in RKO p53i and in T98G cells, compared to RKO-C cells, were not further increased by cobalt treatment, at least at the time point analyzed; however, they were strongly suppressed by HIPK2 overexpression (Fig. 2B). Altogether, these results indicate that HIPK2 inhibited HIF-1 transcriptional activity, in cobalt-treated cells, suppressing MDR1 and Bcl2 gene expression.

### Effect of HIPK2 on cell death and survival after cobalt treatment

**Figure 3:**
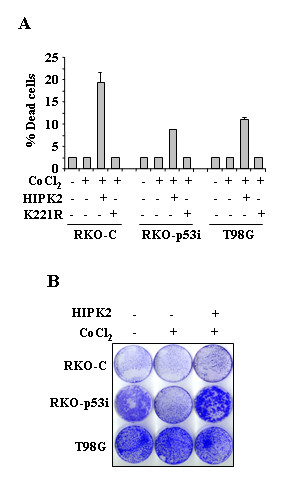
**Effect of HIPK2 on cell death and survival after cobalt treatment**. RKO-C, RKO-p53i, and T98G cells were transfected with HIPK2-GFP or K221R-GFP expression vectors. Twelve hours after transfection cells were treated with CoCl_2 _(200 μM) for 24 h. Thirty-six hours after transfection cells cell viability was assayed by Trypan blue exclusion. The results shown are representative of three independent experiments performed in duplicate. (B) Colony-forming ability of RKO-C, RKO-p53i, and T98G cells transfected and treated as in (A). Death-resistant colonies were stained with crystal violet 14 days after transfection.

Next we evaluated whether HIPK2 overexpression could affect cell death and survival of cobalt-treated cells. To this aim, RKO-C, RKO-p53i and T98G cells were transfected with HIPK2-GFP and K221R-GFP expression vectors and soon after transfection treated with cobalt. The GFP detection by fluorescence microscope allowed to monitor transfection efficiency that was comparable between HIPK2 and K221R expression vectors and among the three cell lines. Cell viability was then determined by Trypan blue exclusion 24 h later. As shown in Fig. 3A, HIPK2 induced cell death, compared to K221R, in cobalt-treated cells, ranging from 10% (in p53i and T98G cells) to 20% (in RKO-C cells). However, long-term analysis of cell survival show that HIPK2 overexpression did not reduce colony-forming ability of the cobalt-treated cells (Fig. 3B). The effect of hypoxia-mimicking condition on survival after HIPK2 overexpression was highly reproducible in three different experiments. We have previously shown that HIPK2 strongly decreases colony formation of RKO cells compared to K221R transfection, inducing p53-dependent apoptosis, furthermore, HIPK2 does not suppress colony formation in either p53 mutant or null cells [2]. Therefore, these data suggest that HIPK2 was able to induce transient cell death in cobalt-treated cells, thus, it did not induce long-lasting cell death, as seen by colony assay, neither apoptosis (see below).

### HIPK2 sensitizes chemoresistant cells to ADR-induced apoptosis in hypoxia-mimicking condition

**Figure 4:**
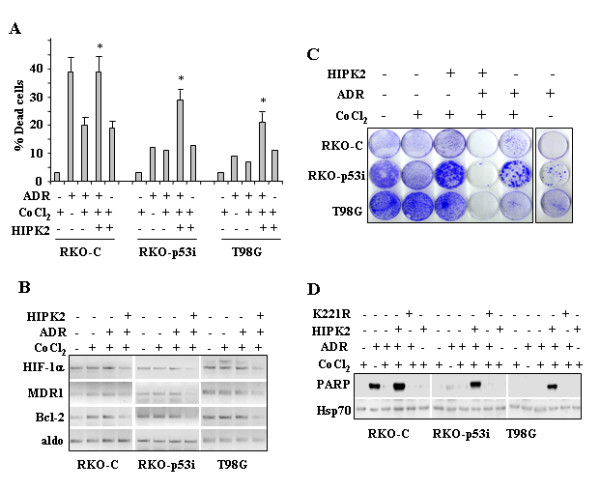
**HIPK2 sensitized resistant cells to ADR-induced apoptosis, in hypoxia-mimicking condition**. (A), RKO-C, RKO-p53i, and T98G cells were transfected with HIPK2-GFP, and the empty GFP vector. Twelve hours after transfection cells were pre-treated with CoCl_2 _(200 μM) for 8 h and then treated with and ADR (2 μg/ml) for 24 h (Total time treatments: 36 h transfection, 24 h ADR; 32 h CoCl_2_). Both floating and adherent cells were collected and cell viability was determined by Trypan blue exclusion. The results shown are representative of three independent experiments performed in duplicate. *p 0.001. (B), RKO-C, -p53i, and T98G cells were treated as in (A) and nuclear cell extracts analyzed by Western immunoblotting with anti-PARP antibody. The cleaved form of PARP is shown. Anti-Hsp70 was used as protein loading control. (C), RKO-C, RKO-p53i, and T98G cells transfected and treated as in (A) were analyzed by RT-PCR analysis for HIF-1α, MDR1, and Bcl2 expression. Aldolase (aldo) was used as loading control. One representative experiment from three independent experiments was shown. (D), Colony-forming ability of RKO-C, RKO-p53i, and T98G cells transfected with HIPK2-GFP and pre-treated with CoCl_2 _for 22 h and ADR (2 μg/ml) for 2 h (Total time treatments: 24 h CoCl_2 _and 2 h ADR). Death-resistant colonies were stained with crystal violet 10 days after transfection.

In the previous paragraphs we have shown that HIPK2 might change the transcriptional potential of HIF-1 in cobalt-treated cells although it did not induce long-term cell death. Therefore, we asked whether HIPK2 could sensitize cobalt-treated cells to drug-induced apoptosis. To this aim, RKO-C, RKO-p53i, and T98G cells were transfected with HIPK2 expression vector; soon after transfection cells were trypsinized, counted and equal cell numbers were re-plated in triplicate prior to the exposure of cells to CoCl_2 _and ADR alone or in combination. Twenty-four hours later, cell viability was assayed by Trypan blue exclusion. As shown in Fig. 4A, exposure to CoCl_2 _strongly reduced the ADR-induced cell death of RKO-C cells, while did not further change the viability of ADR-treated RKO-p53i and T98G cells. Interestingly, HIPK2 overexpression significantly restored the ADR-induced cell apoptosis in cobalt-treated RKO-C cells, as shown by cell viability (Fig. 4A) and Western immunoblotting of cleavage of the caspase-3 substrate PARP (Fig. 4B). It is noteworthy that HIPK2 significantly sensitized to ADR-induced apoptosis also the RKO-p53i and T98G cells (Fig. 4A, 4B) that showed an inherent ADR resistance in anoxic condition (Fig. 4A) and also resistance to HIPK2-induced long-term cell death (Fig. 3B). Moreover, semiquantitative RT-PCR analysis of mRNA levels showed that ADR treatment did not change the HIF-1α, MDR1, and Bcl2 gene expression in cobalt-treated RKO-C, p53i, and T98G cells, on the other hand, HIPK2 overexpression strongly reduced HIF-1α, MDR1, and Bcl2 gene expression (Fig. 4C). The biological outcome of HIPK2 overexpression in cobalt-treated cells, in the presence or absence of ADR was further evaluated by colony forming assay. To this aim, RKO-p53i and T98G cells were transfected with HIPK2 expression vector; soon after transfection cells were treated with cobalt (200 μM) for 24 h before adding 2 h pulse of ADR (2 μg/ml). Cells were then trypsinezed, counted and equal number of cells were re-plated in duplicate in fresh medium and kept in culture for at least ten days. As shown in Fig. 4D, colony forming ability of RKO-C, p53i, and T98G cells treated with combination of cobalt and ADR was markedly suppressed by HIPK2 overexpression, while ADR only partially limited cell growth of cobalt-treated cells. ADR alone strongly suppresses colony formation only in RKO-C cell, as expected (Fig. 4D). Altogether, these data strongly suggest that HIPK2 could reverse cobalt-induced cell chemoresistance of wild-type p53 RKO-C cells and sensitize chemoresistant RKO-p53i and mutant p53 T98G cells to ADR-induced apoptosis. This biological outcome correlated with suppression of MDR1 and Bcl2 gene expression.

## Discussion

Our studies provide a rationale for the potential use of HIPK2 transduction to sensitize chemosensitive tumor cells to ADR-induced apoptosis. We show that HIPK2 downregulated HIF-1α levels in hypoxia-mimicking condition, suppressing HIF-1 transcriptional activity. Thus, HIF-1 target genes such as MDR1 and Bcl-2 were downmodulated by HIPK2 overexpression in cobalt-treated cells: downmodulation of HIF-1α, MDR1, and Bcl2 correlated with ADR-induced apoptosis. Thus, HIPK2 overexpression did not induce per se apoptosis of cobalt-treated cells, on the contrary it sensitized cobalt-treated cells to ADR-induced apoptosis, regardless of their p53 status.

We have previously documented the role of HIPK2 in drug-induced apoptosis. HIPK2 activation by chemotherapeutic drugs, including ADR and cisplatin, results in p53 phosphorylation at Ser46 with specific induction of apoptosis [4,5]; on the other hand, HIPK2 is unable to induce apoptosis in p53 mutant cells [2], although p53-independent apoptotic pathways activated by HIPK2 have been reported [23-25]. We have recently shown that HIPK2 deregulation is involved in chemoresistance however, HIPK2 overexpression circumvents the blockade of apoptosis in chemoresistant cells, restoring activation of p53Ser46 and the caspases' pathway [7]. Therefore, lack of functional p53 contributes to resistance to HIPK2 apoptotic function although, deregulation of the upstream signaling pathways and of the regulatory proteins that finally lead to HIPK2 activation in response to different stimuli cannot be excluded.

In the present study, we have shown that the exogenous HIPK2 might enhance the ADR-apoptotic response impaired by hypoxia-mimicking condition. It is noteworthy that HIPK2 sensitized to ADR-induced apoptosis also the RKO-p53i and T98G cells (carrying mutant p53) that showed an inherent ADR resistance in anoxic condition (Fig. 4A) and also resistance to HIPK2-induced long-term cell death (Fig. 3B). These data suggest that HIPK2 might induce fundamental alterations in cell signaling pathways, involving or not p53-dependent apoptotic function. In this regard, we have recently found that HIPK2 downmodulates HIF-1α expression in normoxic condition, repressing HIF-1 transcriptional activity [9]. HIF-1α is a negative factor for tumor therapy that has a powerful role in modulating chemotherapeutic responses [26]. The expression of multidrug resistance (MDR) via HIF-1-dependent regulation has been shown to increase chemoresistance [21]. Our results showed that overexpression of HIPK2 downmodulated HIF-1α expression and HIF-1 transcriptional activity, in hypoxia-mimicking condition. Blocking of HIF-1α expression abolished MDR1 and Bcl2 expression and restored ADR-induced apoptosis. Reversal of MDR1 and Bcl2 upregulation could therefore provide the mechanistic basis for chemosensitization observed during HIPK2 transduction.

In summary, this study shows the efficacy of HIPK2 transduction to restore the apoptosis-inducing potential of chemotherapeutic drug in hypoxia-mimicking condition and therefore to sensitize chemoresistant tumor cells, regardless of their p53 status.

## Conclusion

We present here a potential approach of combining HIPK2 transduction with chemotherapeutic drug for the treatment of chemoresistant tumor cells. Specifically, HIPK2 restored the apoptosis-inducing potential of chemotherapeutic drug in hypoxia-mimicking condition and sensitized chemoresistant tumor cells. These data suggest that HIPK2 might induce fundamental alterations in cell signaling pathways, involving or not p53 function and provide a foundation for the development of combined regimens that would enhance the apoptotic response of chemoresistant cells. Thus potential use of HIPK2, by either gene therapy deliver or identification of small peptides able to activate HIPK2, is promising for cancer treatment by potentiating cytotoxic therapies, regardless of p53 cell status.

## Methods

### Cell cultures and treatments

Human colon carcinoma RKO (carrying wtp53), RKO-p53-interfered (p53i) (a kind gift of Silvia Soddu, Regina Elena Cancer Institute, Rome Italy), the human glioblastoma T98G (mutant p53, codon 237 methionine-to-isoleucine change) [27], and human lung adenocarcinoma H1299 cells were cultured in RPMI-1640 (GIBCO-BRL, Life Technology, Grand Island, NY, USA) supplemented with 10% heat-inactivated fetal bovine serum (GIBCO-BRL) plus glutamine and antibiotics in humidified atmosphere with 5% CO_2 _at 37°C.

Adriamycin (ADR) was diluted into the medium to a final concentration of 2 μg/ml that we have shown can induce apoptosis in wild-type bearing cells but not in p53 inactive cells [4], for 16-24 h; hypoxia-mimicking condition was obtained by adding cobalt chloride (CoCl_2_) into the culture medium to a final concentration of 200 μM [16] for 24 h; cycloheximide (CHX), the inhibitor of protein synthesis, was diluted into the medium to a final concentration of 40 μg/ml for 3 h.

### RNA extraction and semiquantitative reverse-transcriptase (RT)-PCR analysis

Cells were transfected with HIPK2-GFP, K221-GFP coding HIPK2 kinase defective mutant [2], and the empty GFP vector using LipofectaminePlus method. Soon after transfection cells were trypsinized, counted and equal cell number re-plated in 60 mm Petri dishes. Twelve hours after transfection cells were pre-treated with CoCl_2 _(200 μM) for 8 h and then treated with and ADR (2 μg/ml) for 16 h. Thirty-six hours after transfection, cells were harvested in TRIzol Reagent (Invitrogen) and total RNA was isolated following the manufacturer's instructions. The first strand cDNA was synthesized according to the manufacturer's instructions (Moloney murine leukemia virus reverse transcriptase kit, Applied). Semi-quantitative RT-PCR was carried out by using HOT-MASTER Taq (Eppendorf) with 2 μl cDNA reaction and genes specific oligonucleotides under conditions of linear amplification. The sequence of the primers used for RT-PCR was as follow: HIF-1α forward: 5'-CAGAAGATACAAGTAGCCTC-3'; HIF-1α reverse: 5'-CTGCTGGAATACTGTAACTG-3'; MDR1 forward: 5'-AACGGAAGCCAGAACATTCC-3'; MDR1 reverse: 5'-AGGCTTCCTGTGGCAAAGAG-3'; Bcl-2 forward: 5'-AGGA TTGTGGCCTTCTTTGAG-3'; Bcl-2 reverse: 5'-GAGACAGCCAGGAGAAATCAAA-3'. DNA products were run on 2% agarose gel and visualized by ethidium bromide using UV light. Densitometric analyses was applied to quantify mRNA levels. Data presented are representative of at least three independent experiments.

### Viability assay and colony assay

Cells were transfected with HIPK2-GFP, K221-GFP and the empty GFP vector using LipofectaminePlus method (Invitrogen Corp., Carlsband, CA, USA). Soon after transfection cells were trypsinized, counted and equal cell number re-plated in triplicate in 60 mm Petri dishes. Twelve hours after transfection cells were pre-treated with CoCl_2 _(200 μM) for 8 h and then treated with and ADR (2 μg/ml) for 24 h. Both floating and adherent cells were collected and cell viability was determined by Trypan blue exclusion by direct counting with a haemocytometer, as reported [5].

For colony-formation assay, cells were transfected with expression vectors as above and soon after transfection treated with CoCl_2 _(200 μM) for 22 h before adding a pulse of ADR (2 μg/ml) for 2 h. After treatments, cells were washed, trypsinized, counted and equal cell number re-plated in duplicate with fresh medium, in 60 mm Petri dishes. Death-resistant colonies were stained with crystal violet 10-4 days later. Data presented are representative of at least three independent experiments.

### Transfection, plasmids, and transactivation assay

Transient transfection was carried out using H1299 cells and the cationic polymer LipofectaminePlus method (Invitrogen Corp., Carlsband, CA, USA) according to manufacturers' instructions. The amount of plasmid DNA was equalized in each sample by supplementing with empty vector. Cells were transiently co-transfected with HIPK2-GFP or K221R-GFP expression vectors along with the luciferase reporter gene driven by human erythropoietin enhancer region containing a functional HIF-1 binding site (pEpoE-luc) or a mutant HIF-1 binding site (pEpoE-mut-luc) [15] (kindly provided by L. Eric Huang, N.C.I., N.I.H, Bethesda, MA, USA). Soon after transfection cells were trypsinized, counted and equal cell number re-plated in 60 mm Petri dishes. Twelve hours after transfection cells were pre-treated with CoCl_2 _(200 μM) for 8 h and then treated with and ADR (2 μg/ml) for 16 h. Transfection efficiency was normalized with the use of a co-transfected β-galactosidase (β-gal) plasmid. Thirty-six hours after transfection, cells were harvested and luciferase activity was assayed on whole cell extract. Luciferase values were normalized to β-galactosidase activity and protein content. At least three independent experiments were performed in duplicate.

### Western blot analysis

Total cell extracts were prepared by incubating at 4°C for 30 min in lysis buffer (50 mM Tris-HCl, pH 7.5, 50 mM NaCl, 5 mM EDTA, 150 mM KCl, 1 mM dithiothreitol, 1% Nonidet P-40) plus a mix of protease and phosphatase inhibitors (Sigma Chemical Company, MO, USA). Nuclear extracts were prepared essentially as described [28]. Proteins were then separated by 9% SDS-PAGE and blotted onto nitrocellulose (BioRad, CA, USA). The membranes were probed with a primary antibody followed by with horseradish-peroxidase conjugated secondary antibody. The antibodies used were: monoclonal anti-HIF-1α (Novus Biologicals, UCS Diagnostic, Italy), monoclonal anti-poly(ADP-ribose) polymerase (PARP, BD Pharmingen, CA, USA), and monoclonal anti-Hsp70 (Stressgene, BC, Canada). Immunoreactivity was detected with the Advanced-ECL chemoluminescence reaction kit (Amersham., IL, USA).

### Statistics

Continuous variables were analyzed by the Student *t *test. Data are expressed as mean ± SD. A value of p ≤ 0.05 was considered statistically significant.

## Abbreviations

ADR: adriamycin; RT-PCR: reverse-transcriptase-PCR; HIF-1: Hypoxia-inducible factor; HRE: hypoxia response elements; MDR1: multidrug resistance; PARP: poly(ADP-ribose) polymerase; CoCl_2_: cobalt chloride; CHX: cycloheximide.

## Competing interests

The authors declare that they have no competing interests.

## Authors' contributions

LN and RP performed the experiments. AS supervised the project. GDO designed the experiments, supervised the project, and prepared the manuscript. All authors read and approved the final manuscript.

## References

[B1] Kim YH, Choi CY, Lee SJ, Conti MA, Kim Y (1998). Homeodomain-interacting protein kinases, a novel family of co-repressors for homeodomain transcription factors. J Biol Chem.

[B2] D'Orazi G, Cecchinelli B, Bruno T, Manni I, Higashimoto Y, Saito S, Gostissa M, Coen S, Marchetti A, Del Sal G, Piaggio G, Fanciulli M, Appella E, Soddu S (2002). Homeodomain-interacting protein kinase-2 phosphorylates p53 at Ser 46 and mediates apoptosis. Nature Cell Biol.

[B3] Hofmann TG, Moller A, Sirma H, Zebtgraft H, Taya Y, Droge W, Will H, Schmitz ML (2002). Regulation of p53 activity by its interaction with homeodomain-interacting protein kinase-2. Nauret Cell Biol.

[B4] Puca R, Nardinocchi L, Gal H, Rechavi G, Amariglio N, Domany E, Notterman DA, Scarsella M, Leonetti C, Sacchi A, Blandino G, Givol D, D'Orazi G (2008). Reversible dysfunction of wild-type p53 following homeodomain-interacting protein kinase-2 knockdown. Cancer Res.

[B5] Di Stefano V, Rinaldo C, Sacchi A, Soddu S, D'Orazi G (2004). Homeodomain-interacting protein kinase-2 activity and p53 phosphorylation are critical events for cisplatin-mediated apoptosis. Exp Cell Res.

[B6] Nardinocchi L, Puca R, Sacchi A, D'Orazi G (2007). HIPK2 knock-down compromises tumor cell efficiency to repair damaged DNA. Biochem Biophys Res Commun.

[B7] Puca R, Nardinocchi L, Pistritto G, D'Orazi G (2008). Overexpression of HIPK2 circumvents the blockade of apoptosis in chemoresistant ovarian cancer cells. Gynecol Oncol.

[B8] D'Orazi G, Sciulli MG, Di Stefano V, Riccioni S, Frattini M, Falcioni R, Bertario L, Sacchi A, Patrignani P (2006). Homeodomain-interacting protein kinase-2 restrains cytosolic-phospholipase-A2-dependent prostaglandin-E2 generation in human colorectal cancer cells. Clin Cancer Res.

[B9] Nardinocchi L, Puca R, Guidolin D, Belloni AS, Bossi G, Michiels C, Sacchi A, Onisto M, D'Orazi G (2008). Trancriptional regulation of hypoxia-inducible factor 1alpha by HIPK2 suggests a novel mechanism to restrain tumor growth. BBA Mol Cell Res.

[B10] Puca R, Nardinocchi L, D'Orazi G (2008). Regulation of vascular endothelial growth factor expression ny homeodomain-interacting protein kinase-2. J Exp Clin Cancer Res.

[B11] Semenza GL (2003). Involvement of hypoxia-inducible factor 1 in human cancer. Nature Rev Cancer.

[B12] Wang GL, Jiang B-H, Rue EA, Semenza GL (1995). Hypoxia-inducible factor 1 is a basic-helix-loop-helix-PAS heterodimer regulated by cellular O2 tension. Proc Natl Acad Sci USA.

[B13] Jiang BH, Semenza GL, Bauer H, Marti H (1996). Hypoxia-inducible factor 1 levels vary exponentially over a physiologically relevant range of O2 tension. Am J Physiol.

[B14] Liu L, Ning X, Sun L, Zhang H, Shi Y, Guo C, Han S, Liu J, Sun S, Han Z, Wu K, Fan D (2008). Hypoxia-inducible factor-1a contributes to hypoxia-induced chemoresistance in gastric cancer. Cancer Sci.

[B15] Huang LE, Arany Z, Livingston DM, Bunn HF (1996). Activation of hypoxia-inducible transcription factor depends primarily upon redox-sensitive stabilization of its α subunit. J Biol Chem.

[B16] Wang GL, Semenza GL (1993). General involvement of hypoxia-inducible factor 1 in transcriptional response to hypoxia. Proc Natl Acad Sci USA.

[B17] Raguz S, Yague E (2008). Resistance to chemotherapy: new treatments and novel insights. Br J Cancer.

[B18] Greijer AE, Wall E van der (2004). The role of hypoxia inducible factor 1 (HIF-1) in hypoxia induced apoptosis. J Clin Pathol.

[B19] Borst P, Jankers J, Rottenberg S (2007). What makes tumors multidrug resistant?. Cell Cycle.

[B20] Fardel O, Lecureur V, Guillouzo A (1996). The P-glicoprotein multidrug transporter. Gen Pharmacol.

[B21] Comerford KM, Wallace TJ, Karhnausen J, Louis NA, Montalto MC, Colgan SP (2002). Hypoxia-inducible factor-1-dependent regulation of the multidrug resistance (MDR1) gene. Cancer Res.

[B22] Talks KL, Turley H, Gatter KC, Maxwell PH, Pugh CW, Ratcliff PJ, Harris AL (2000). The expression and distribution of the hypoxia-inducible factors HIF-1alpha and HIF-2alpha in normal human tissues, cancers, and tumor-associated macrophages. Am J Pathol.

[B23] Zhang Q, Yoshimatsu Y, Hildebrabd J, Frisch SM, Goodman RH (2003). Homeodomain interacting protein kinase 2 promotes apoptosis by downregulating the transcriptional corepressor CtBP. Cell.

[B24] Hofmann TG, Stollberg N, Schmitz ML, Will H (2003). HIPK2 regulates transforming growth factor-beta-induced c-Jun NH(2)-terminal kinase activation and apoptosis in human hepatoma cells. Cancer Res.

[B25] Doxakis E, Huang EJ, Davies AM (2004). Homeodomain-interacting protein kinase-2 regulates apoptosis in developing sensory and sympathetic neurons. Curr Biol.

[B26] Unruh A, Ressel A, Mohamed HG, Johnson RS, Nadrowitz R, Richter E, Katschinski DM, Wenger RH (2003). The hypoxia-inducible factor-1α is a negative factor for tumor therapy. Oncogene.

[B27] Chen P, Iavarone A, Fick J, Edwards M, Prados M, Israel MA (1995). Constitutional p53 mutations associated with brain tumors in young adults. Cancer Genetics and Cytogenetics.

[B28] Di Stefano V, Mattiussi M, Sacchi A, D'Orazi G (2005). HIPK2 inhibits both MDM2 gene and protein by, respectively, p53-dependent and independent regulations. FEBS Lett.

